# Deformation Response of the Human Lamina Cribrosa to Intracranial Pressure Lowering

**DOI:** 10.1115/1.4068633

**Published:** 2025-07-01

**Authors:** Kelly A. Clingo, Cameron A. Czerpak, Sara Grace Ho, Megha Patel, Crystal Favorito, Anny Zheng, Abhay Moghekar, Harry A. Quigley, Thao D. Nguyen

**Affiliations:** Department of Mechanical Engineering, The Johns Hopkins University, Baltimore, MD 21218; Department of Neurology, Johns Hopkins School of Medicine, Johns Hopkins University, Baltimore, MD 21287; Johns Hopkins School of Medicine, Wilmer Eye Institute, Baltimore, MD 21287; Department of Mechanical Engineering, The Johns Hopkins University, Baltimore, MD 21218; Johns Hopkins School of Medicine, Wilmer Eye Institute, Baltimore, MD 21287; Johns Hopkins University; Johns Hopkins University; Johns Hopkins Medicine; Johns Hopkins Medicine; Johns Hopkins Hospital; Johns Hopkins University; Johns Hopkins Medicine

**Keywords:** intracranial pressure, glaucoma, optic nerve head, biomechanics, space-associated neuro-ocular syndrome

## Abstract

The optic nerve head (ONH) is subjected to both intra-ocular pressure (IOP) and intracranial pressure (ICP). The translaminar pressure difference (TLPD) is defined as the difference between IOP and ICP. A change in TLPD, whether from changes in IOP or ICP, could subject the lamina cribrosa (LC) to altered deformation, potentially damaging the axons, activating the mechanosensitive glial cells, and promoting remodeling of the connective tissue structures in the ONH. In this study, we applied spectral domain optical coherence tomography (SD-OCT) and digital volume correlation (DVC) to calculate the deformation response of the LC in 7 eyes of 7 patients with normal pressure hydrocephalus (NPH). Radial SD-OCT scans centered on the ONH were acquired prior to and after therapeutic extended cerebrospinal fluid (CSF) drainage. IOP was measured immediately before imaging, and ICP was measured at the beginning and end of the drainage procedure. The procedure led to a mean ICP decrease of 11.24±1.84 mmHg and a small, nonsignificant mean IOP increase of 0.67±2.56 mmHg. ICP lowering produced a significant $E_{zz}=-0.50\%\pm 0.47\%$, $E_{rr}=0.53\%\pm 0.48\%$, and $E_{\theta z}=0.35\%\pm 0.21\%$ ( p≤0.031). A larger compressive $E_{zz}$ was associated with a larger ICP decrease ( p=0.007). Larger $E_{rr}$, $E_{r\theta }$, maximum principal strain, $E_{\max}$ and maximum shear strain, $S_{\max}$ in the plane of the radial scans were associated with a larger increase in a calculated TLPD change ( p≤0.035).

## 1 Introduction

The interaction between intra-ocular pressure (IOP) and intracranial pressure (ICP) plays an important role in glaucoma and other eye diseases, including space-associated neuro-ocular syndrome. IOP deforms the optic nerve head (ONH) both directly as a normal traction on the anterior surface of the ONH and indirectly by inducing tensile hoop stresses in the sclera, which then exert normal and shear traction on the lateral boundary of the ONH. ICP impinges on the sclera only in the narrow subarachnoid space of the optic nerve sheath, which is contiguous with the subarachnoid space of the brain and spinal cord. The ICP in the subarachnoid space also determines the retrolaminar tissue pressure (RLTP) that acts normal to the posterior surface of the ONH. Morgan et al. [1] measured the optic nerve tissue pressure through the thickness of the lamina cribrosa (LC) in dogs and showed that the RLTP was about 80% of the ICP for ICP greater than 1.3 mmHg, and was 3.7 mmHg at zero ICP.

There is some evidence that lower ICP is a risk factor for glaucoma [2,3]. Various retrospective studies of patients who underwent lumbar puncture showed that ICP was significantly lower in patients with primary open angle glaucoma (POAG) and normal tension glaucoma (NTG) than in age-matched control subjects without glaucoma [4,5]. More recent prospective studies have corroborated these findings [6,7], though a few studies have reported no differences in the ICP of NTG and normal subjects [8,9]. NPH patients who received shunts to lower ICP also had a higher incidence of NTG [10,11]. The difference between the IOP and ICP is the TLPD. A higher TLPD, from either elevated IOP or reduced ICP, may subject the LC to altered deformation, causing injury to the axons coursing through the LC. Yang et al. [12], showed that lowering ICP in monkeys using a shunt produced retinal ganglion cell axon loss in the neuroretinal rim in 2 of 4 monkeys.

Experimental and computational modeling studies have started to examine the effects of ICP change on the deformation of the LC. Optical coherence tomography (OCT) and DVC have been applied to measure the deformation response of the LC in monkeys [13–16] and human eyes of a brain-dead organ donor [17] to controlled IOP and ICP changes. Changing IOP or ICP produced local regions of large tensile, compressive, and shear strains in the LC. The strain response varied significantly between different eyes, and the sample size of the studies has been too small to discern statistically significant patterns in the strain field or significant relationships between the LC strain response and pressure changes. Lowering ICP did not produce the opposite effect as lowering IOP, and the LC deformation response to ICP change depended on the level of IOP [16,18]. Computational modeling studies have also confirmed the important effects of ICP and IOP on the LC strain response [19–22]. However, these models suggest that LC deformation was more sensitive to IOP change than ICP change [22,23].

In this study, we applied spectral domain optical coherence tomography (SD-OCT) imaging and DVC to characterize the LC deformation response of NPH patients to ICP lowering by extended CSF drainage. We applied the same imaging and image processing methods developed previously to characterize the ONH deformation response to IOP change by suturelysis and glaucoma medication [24–26], and examined the reproducibility of the DVC strain calculation for ICP change. Finally, we examined the relationship between the LC strain response and the IOP, ICP, and TLPD change.

## 2 Methods

### 2.1 Experimental Subjects.

**Fig. 1:**
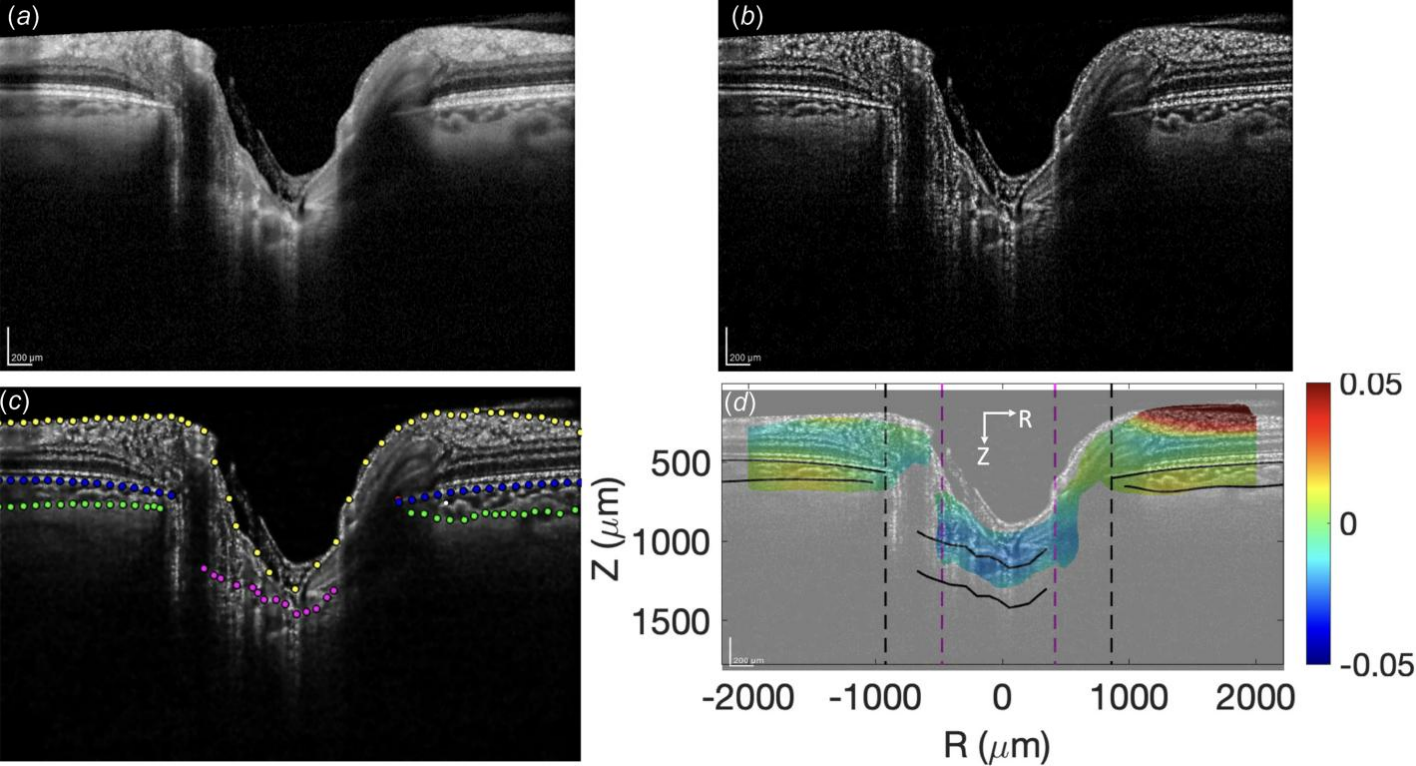
The postprocessing of SD-OCT image volumes for DVC calculation, showing a radial scan (*a*) acquired after ICP lowering, (*b*) after contrast enhancement using contrast-limited adaptive histogram equilization and a Gamma filter, and (*c*) after segmentation of the tissue boundaries using a trained convolution neural network model. (*d*) A color contour map of the $E_{zz}$ response to ICP lowering plotted on top of the SD-OCT radial scan.

This study was approved by the Institutional Review Board of the Johns Hopkins School of Medicine. Written informed consent was obtained from all patients prior to IOP measurement and SD-OCT imaging. We successfully imaged the ONH of 17 eyes from 9 NPH patients by SD-OCT (Spectralis, Heidelberg Engineering) at the Cerebrospinal Fluid Center, Johns Hopkins School of Medicine, immediately before and after an extended CSF drainage procedure, during which 300–400 ml of CSF is drained over 2 days. Extended CSF drainage is a standard diagnostic test to determine whether NPH patients would be responsive to a more permanent shunt [27,28]. The ages of the patients ranged from 45 to 81 years (mean: 66.6±11.1 years, median: 69 years), and one eye was diagnosed with open angle glaucoma (OAG). On the day of the procedure and before the lumbar puncture, axial length was measured by IOL Master (Zeiss, Dublin, CA), IOP was measured by iCare tonometer (iCare Finland Oy, Espoo, Finland), and three sets of radial SD-OCT scans were acquired with the patients sitting at the bedside (Sec. 2.2, Fig. 1(*a*)). For the CSF drainage procedure, a catheter was inserted into the lumbar spine, and the initial CSF opening pressure was recorded. After the completion of the CSF drainage procedure, the ICP was remeasured, and the IOP measurement and SD-OCT imaging was repeated with the patient sitting at the bedside 20 min after catheter removal.

Digital volume correlation was applied to the pre- and post-treatment images to calculate the deformation response of the LC as described in Sec. 2.4. However, accurate DVC displacement calculations were obtained in less than 20% of the LC for 7 eyes, which suffered from poor contrast and were omitted from further analysis. The remaining eyes included 4 right eyes (OD) from 4 subjects and both eyes from an additional 3 subjects. The left and right eyes of the same individual were associated with the same ICP measurement. Thus, including both eyes of the same individual would increase the variability in the relationships between the measured outcomes and ICP. The small sample size precluded statistical analysis considering the correlation between the left and right eyes of the same subject. Thus, we excluded the 3 left eyes (OS) in this initial analysis. The remaining 7 right eyes of 7 patients included the one OAG eye and had the same age range (mean: 66.4.±11.9 years), similar baseline IOP, baseline ICP, ICP change, and IOP change as the larger group of 17 eyes.

### 2.2 Imaging and Image Processing.

A volume of 24 radial SD-OCT scans was acquired centered on the ONH using the Spectralis ONH-RC program (Fig. 1). The radial scans were 768 × 495 pixels in (R,Z), which corresponded to 3.87 μm/pixel in *Z* and 5.52–6.15 μm/pixel in *R* depending on the measured axial length. The 24 radial scans were separated by 7.5 deg between adjacent scans, resulting in a 108 μm/pixel resolution in the circumferential ( Θ) direction at Bruch's membrane opening. The ONH-RC program utilizes the center of the BMO and the fovea of each eye to orient the radial scan pattern. Thus, the radial scan volumes of the same eye taken pre- and post-treatment were registered to the same eye-specific anatomic landmark. We did not perform additional registration of the pre- and post-treatment images. The image contrast of the radial scan was enhanced by contrast-limited, adaptive histogram equalization in FIJI [29]. A gamma correction of 1.75 was applied to the image histogram to reduce noise. The image processing settings were optimized to minimize the DVC baseline and correlation error, as described in prior studies [24–26]. The enhanced images were imported into MATLAB 2023a (Mathworks, Natick, MA) and reconstructed as a matrix of 8-bit intensity values for DVC.

### 2.3 Image Segmentation.

Bruch's membrane (BM), Bruch's membrane opening (BMO), the choroidal-scleral interface, and the anterior border of the LC were identified on the pretreatment radial SD-OCT scans using a convolutional neural network described in Clingo et al. [30] (Fig. 1(*c*)). The convolutional neural network was trained on manual markings of the tissue boundaries on the radial SD-OCT scans following contrast enhancement. The BMO was marked on each radial scan as the rightmost end of the left side of the BM and the leftmost end of the right side of the BM, which appears as a bright line at the retina-choroid interface. The lateral borders of the LC were defined by extending vertical lines (in *Z*) from each BMO end position. The posterior border of the LC was defined by a parallel curve placed 250 μm posterior to the anterior border.

### 2.4 Digital Volume Correlation.

The Fast Iterative DVC (FI-DVC) algorithm, developed by Bar-Kochba et al. [31], was applied to analyze the enhanced pre- and post-treatment SD-OCT images to calculate the deformation response of the ONH tissues to ICP change [26,32,33]. Briefly, the FI-DVC method assumes that the displacement mapping from the reference to deformed images can be decomposed additively into *k* increments: $u_{k}(x)=u_{k-1}(x)+du_{k}(x)$. For each iteration, a fast fourier transform implementation of the cross-correlation formulation is used to calculate the displacement increment, and the resulting updated displacement mapping is used to warp the reference and deform images closer together. In the following iterations, the subset size is halved, and the cross-correlation formulation is applied again to the warped images to calculate the next displacement increment. The process is repeated until the convergence criterion for the sum-squared difference between the intensity patterns of the two images is satisfied. Midgett et al. [24] modified the FI-DVC implementation to analyze a cylindrical volume of radial SD-OCT scans rather than a rectangular array of scans and calculate the displacement and strain components in a cylindrical coordinate system centered about the ONH. Midgett et al. [24] also determined the optimal FI-DVC settings to minimize the DVC baseline and correlation errors. These include a 256 × 256 × 32 pixels starting subset size in (R, Z, Θ), 2 × 2 × 1-pixel minimum subset size, 1 × 1 × 1 pixels grid spacing, 0.055 correlation coefficient threshold, and 0.0625 global convergence criteria for the current sum-squared error between the image intensity patterns of the two images normalized by the initial error.

The DVC calculated displacement field was filtered using a 0.055 correlation coefficient threshold as defined in Bar-Kochba et al. [31] to remove these poorly correlated points from the strain calculation. Previous studies showed that a correlation coefficient below 0.055 indicates that the region was too dark and featureless to yield accurate DVC displacement calculations [24,32]. Next, a displacement error filter was applied to remove regions where the DVC displacement correlation errors (Sec. 2.5) exceeded 5 μm for any displacement component. Finally, displacements on the edges of the radial scans and displacement outliers were removed as described in Midgett et al. [24] After applying these displacement filters, the percent LC area of accurate DVC correlation for each eye was calculated as the average over 24 radial scans. Eyes with accurate DVC correlation in less than 20% of the LC area were excluded from further analysis. The average percent LC area of accurate DVC correlation for the remaining eyes was 38.5% ±17.1%.

After applying the displacement filters, the displacement field was smoothed, and the strain components in the cylindrical coordinate system were calculated from the gradient of the smoothed displacement field as described in Midgett et al. [24] The change of the anterior lamina depth (ALD) was calculated in each radial scan from the DVC displacement of the anterior LC surface relative to the BMO as described in Midgett et al. [24], then averaged over the 24 radial scans for each eye.

### 2.5 Digital Volume Correlation Error Estimation.

The DVC baseline and correlation displacement and strain errors were calculated for each eye. The DVC baseline errors were calculated by applying the FI-DVC algorithm to two sets of pretreatment images acquired back-to-back at nominally the same IOP and ICP. The mean baseline strain error for each eye was calculated as the average strain over the volume of accurate DVC correlation in the ONH. The baseline error signifies the error in reproducibility associated with the image quality, including the quality of the natural speckle pattern of the ONH and patient movement during image acquisition.

The DVC correlation error was calculated by numerically warping a pretreatment image by a rigid body translation $U_{Z}=10\mu$m and a uniform 2% strain in *R* and −2% strain in Z. FI-DVC was then applied to the numerically deformed and undeformed images to calculate the displacement and strain fields. The DVC correlation strain errors were calculated as the difference between the numerically applied and DVC calculated displacement and strain fields. The correlation error estimates the effect of distortion of the speckle pattern by the applied deformation on the accuracy of the DVC calculations.

### 2.6 Statistical Analysis.

The outcomes of the DVC calculations for each eye were the strain components in cylindrical coordinates, the maximum principal strain, $E_{\max}$, and the maximum shear strain, $S_{\max}$, in the RZ plane (the plane of the radial scan), and the ALD displacement averaged over the LC. We calculated $E_{\max}$ and $S_{\max}$ in the RZ plane rather than their three-dimensional counterparts because the DVC baseline and correlation displacement errors were largest for the circumferential direction, i.e., for $U_{\Theta }$, which is the scan direction [24]. A paired t-test was applied to analyze the significance of the average strain outcomes and to compare the strains to their corresponding baseline errors. Linear regression was used to analyze the relationship between the strain response and the change in IOP, ICP, and TLPD (defined as IOP-ICP), age, and ALD displacement. Statistical significance was indicated by p<0.05. As described in Sec. 2.1, the statistical analyses were applied to data from 7 right eyes of 7 patients. To investigate the effect of including both eyes from the same patient, we repeated the analyses for 10 eyes of the 7 patients and presented the results in the Supplemental Materials on the ASME Digital Collection.

## 3 Results

### 3.1 Intra-Ocular Pressure Change.

The average baseline IOP and ICP measured before the CSF drainage procedure were 15.83 ±5.11 mmHg and 10.94 ± 1.85 mmHg, respectively, for n=17 eyes of 9 patients. The extended CSF drainage procedure decreased the ICP to zero and was associated with a nonsignificant average increase in IOP of 0.47 ±2.66 mmHg ( p=0.48, n=17). The IOP change was not significantly associated with ICP change ( p=0.12, Fig. 7(*a*)). The smaller group of n=7 eyes from 7 patients had a similar average baseline IOP (17.71 ±5.06 mmHg) and baseline ICP (11.45 ±1.58 mmHg), and experienced a similar mean ICP change (−11.24 ±1.84 mmHg) and mean IOP change (0.67 mmHg ±2.56 mmHg) from extended CSF drainage. The IOP change was also not associated with ICP change for this smaller group of 7 eyes ( p=0.998, Fig. 7(*b*)).

### 3.2 The Strain Response of the Lamina Cribrosa.

**Fig. 2:**
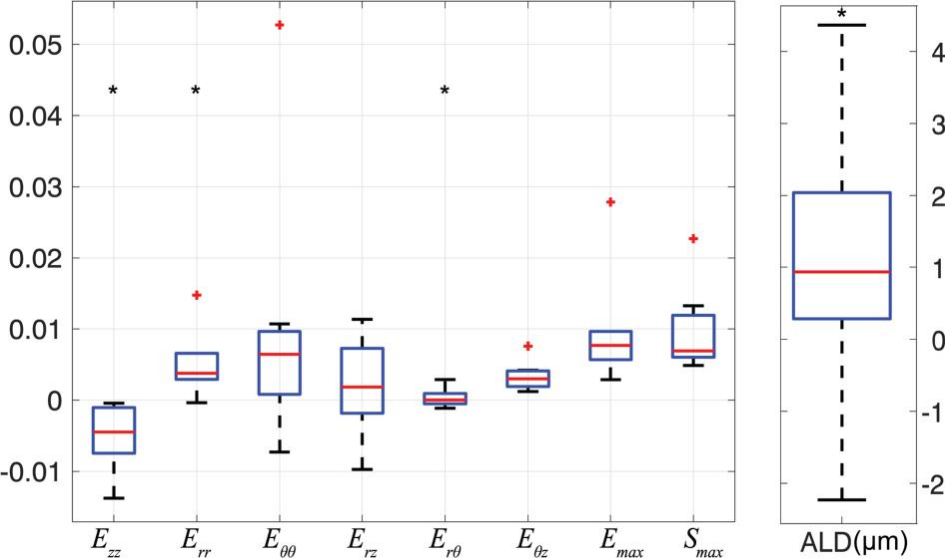
Box plot of the Green–Lagrange strain components and the maximum principal, $E_{\max}$ and maximum shear $S_{\max}$ strains in the plane of the radial scans (RZ) for n=7 eyes, showing the lower and upper quartile, median (−), and outliers (+). Also plotted is the mean ALD change. A positive ALD change signifies a posterior movement of the anterior LC surface. The (*) indicates the mean outcome was statistically nonzero. ICP lowering produced a significant compressive $E_{zz}$ ( p=0.031), tensile $E_{rr}$ ( p=0.025), positive twist shear strain $E_{\theta z}$ ( p=0.005), and a small significant posterior motion of the ALD ( p=0.024).

For the 7 eyes of 7 subjects, the DVC baseline error was statistically significant only for the axial strain component $E_{zz}$ ( p=0.0035), indicating that the DVC calculation of $E_{zz}$ had a small tensile bias (Table 1). The ICP decrease produced a significant compressive axial strain $E_{zz}=-0.0050\pm 0.0047$ ( p=0.031), tensile radial strain $E_{rr}=0.0053\pm 0.0048$ ( p=0.025), and a significant positive shear strain $E_{\theta z}=0.0035\pm 0.0021$ ( p=0.005), indicating a twisting of the ONH (Table 1, Fig. 2). Both $E_{zz}$ and $E_{rr}$ were significantly different than their respective baseline errors ( p<0.05). Interestingly, the magnitude of the radial strain and axial strains were nearly identical, though they had different signs. This indicates that the LC experienced significant shear in the RZ plane in addition to the significant out-of-plane $E_{\theta z}$ shear strain. Indeed, the average maximum shear strain $S_{\max}=0.0097\pm 0.0063$ was as large as the maximum principal strain $E_{\max}=0.0099\pm 0.0083$ in the RZ plane.

**Table 1:** The DVC calculated strains and baseline strain errors.

	Response to ICP		*p*-val	Baseline error		*p*-val
Strains	Mean	Std	Strain ≠ 0	Mean	Std	Strain ≠ error
$E_{zz}$	−5.03 × 10^−3^	4.73 × 10^−3^	**0.031**	1.32 × 10^−3^	7.52 × 10^−4^	**0.015**
$E_{rr}$	5.35 × 10^−3^	4.77 × 10^−3^	**0.025**	4.00 × 10^−4^	6.68 × 10^−4^	**0.049**
$E_{\theta \theta }$	1.04 × 10^−2^	1.95 × 10^−2^	0.21	−3.66 × 10^−4^	9.97 × 10^−4^	0.19
$E_{r\theta }$	1.67 × 10^−3^	7.05 × 10^−3^	0.55	−3.77 × 10^−4^	7.52 × 10^−4^	0.45
$E_{\theta z}$	3.45 × 10^−3^	2.11 × 10^−3^	**0.0050**	−1.93 × 10^−3^	9.08 × 10^−3^	0.12
$E_{rz}$	3.15 × 10^−4^	1.34 × 10^−2^	0.56	2.97 × 10^−4^	5.69 × 10^−4^	0.97

Bold values indicate stastistically significant comparisons, where *p* < 0.05.

### 3.3 Change in Anterior Lamina Depth.

Intracranial pressure lowering produced a small but significant ALD change (mean: 1.67±1.48μm, p=0.024, Fig. 2). A positive ALD change signifies a posterior movement of the anterior LC surface. The ALD change was significantly larger than the baseline error for $U_{z}$ ( $e_{U_{z}}=0.084\pm 0.37\mu$m, p=0.039). ALD change was unrelated to the LC strain response and to IOP change ( p>0.2, Table 2).

### 3.4 Relationships Between Lamina Cribrosa Deformation, Age, and Axial Length.

**Fig. 3:**
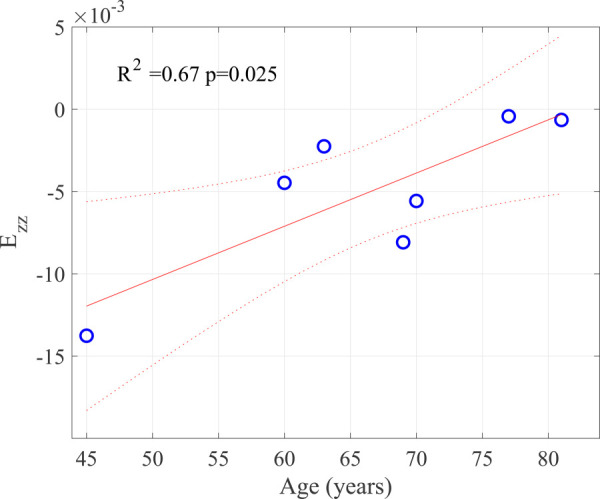
The compressive $E_{zz}$ response is stiffer with older age ( p=0.025)

**Fig. 4:**
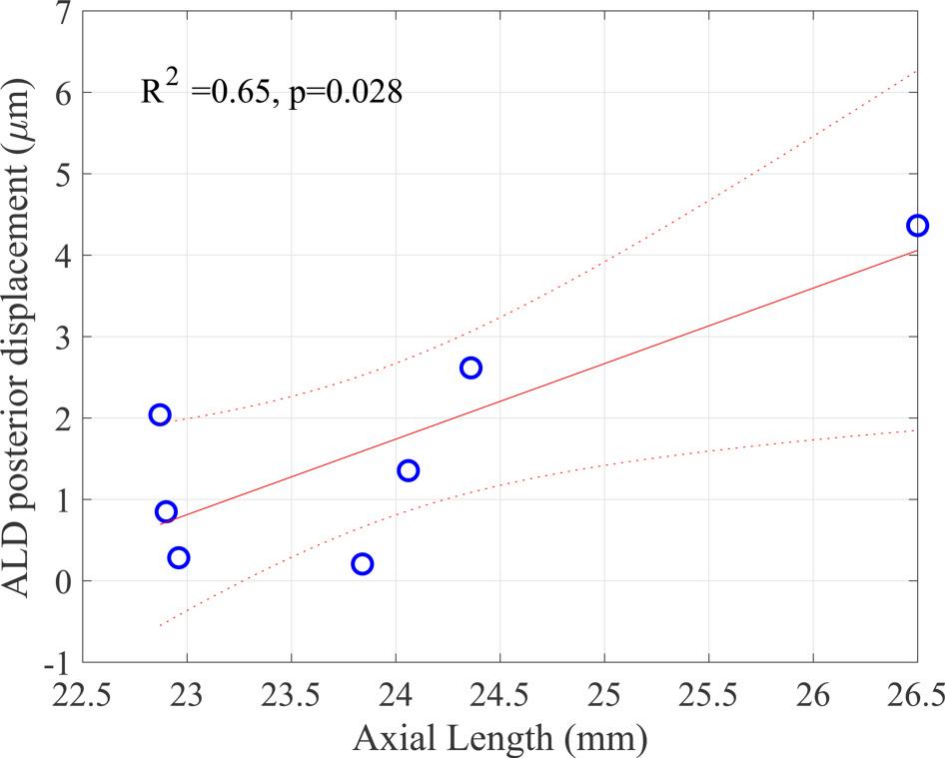
A larger posterior motion of the ALD is associated with greater axial length ( p=0.028)

The compressive $E_{zz}$ response was stiffer for older age ( p=0.025), but the other strain outcomes and ALD change were not significantly related to age ( p>0.09, Fig. 3, Table 3). A larger posterior displacement of the ALD was associated with a larger axial length ( p=0.028, Fig. 4, Table 4), but strains were not related to axial length ( p≥0.3).

### 3.5 Relationships Between the Lamina Cribrosa Strain Response, Intracranial Pressure, Intra-Ocular Pressure, and Translaminar Pressure Difference.

**Fig. 5:**
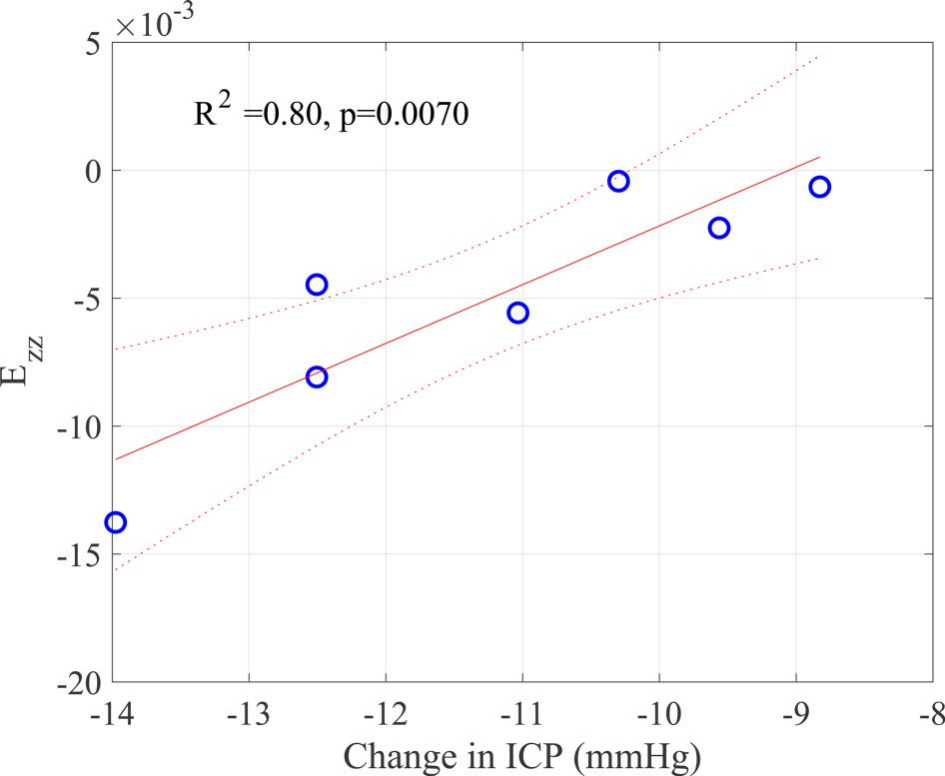
A greater decrease in ICP produced a larger compressive $E_{zz}$ ( p=0.0070)

**Fig. 6:**
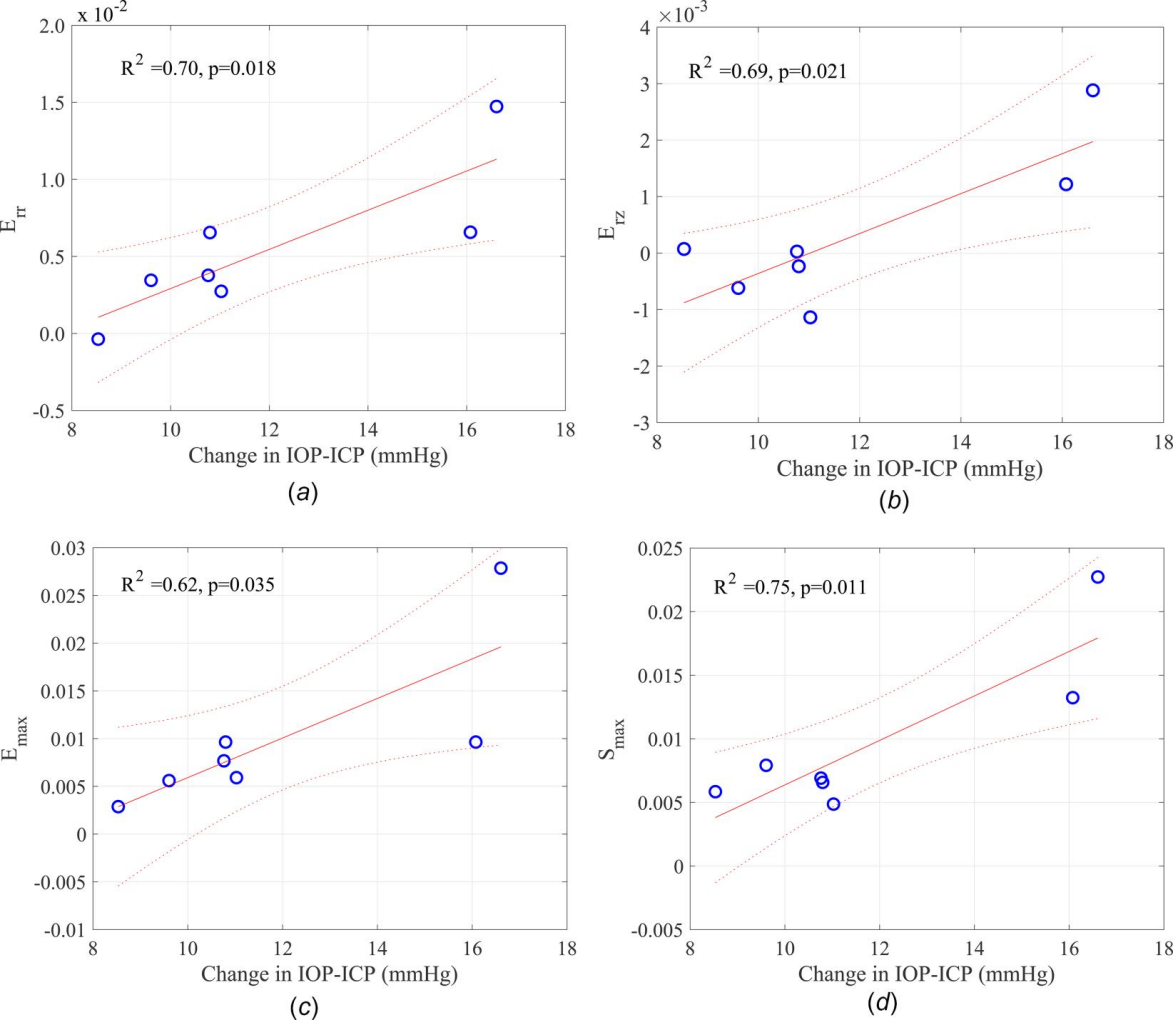
Effect of a TLPD (IOP-ICP) increase on (*a*) $E_{rr}$, (*b*) $E_{rz}$, (*c*) $E_{\max}$, and (*d*) $S_{\max}$. A larger increase in the TLPD produced a significantly larger radial strain $E_{rr}$ ( p=0.018), $E_{rz}$ ( p=0.021), $E_{\max}$ ( p=0.035), and $S_{\max}$ ( p=0.011).

**Fig. 7:**
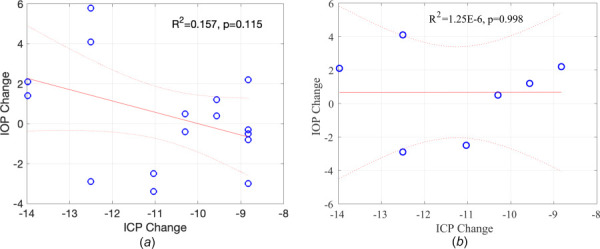
The IOP change was not statistically associated with the ICP change from extended CSF drainage for (*a*) n=17 eyes from nine patients and (*b*) n=7 eyes from seven patients

The LC strain response and ALD change did not vary significantly with the baseline IOP ( p>0.25, Table 5), or with the IOP change ( p>0.06, Table 7). A larger $E_{rr}$ ( p=0.060) and $E_{\max}$ ( p=0.067) were measured for a greater IOP increase; however, the association did not achieve significance. A larger decrease in ICP was associated with a larger compressive $E_{zz}$ response ( p<0.0070, Fig. 5, Table 6). None of the other strain components of the LC strain response nor the ALD change were associated with ICP change ( p>0.13, Table 6). A greater increase in the calculated translaminar pressure difference, defined as TLPD = IOP - ICP, produced a greater $E_{rr}$ ( p=0.018), greater $E_{rz}$ ( p=0.021), greater $E_{\max}$ ( p=0.035), and greater $S_{\max}$ ( p=0.0112) response (Fig. 6, Table 8). The $E_{zz}$ was not associated with the TLPD ( p=0.35).

## 4 Discussion

Digital volume correlation analysis of SD-OCT radial scans can accurately measure the strain response of the LC to ICP lowering after extended CSF drainage in NPH patients. The SD-OCT scans of the ONH for the NPH patients tended to have poorer resolution than those of glaucoma patients in previous studies, possibly due to the expected difficulty in cooperation among recently treated persons. Consequently, DVC correlation was obtained in a smaller percentage of the LC area than in previous studies of POAG patients [25,26]. The baseline strain errors were in line with those in previous studies of IOP lowering in glaucoma patients once samples were compared only in those with accurate DVC correlation in greater than 20% of the LC. The axial strain was significantly more compressive and the radial strain was significantly more tensile than their respective baseline errors.

The NPH patients experienced a mean ICP decrease of 11.24 ±1.79 mmHg, similar in magnitude to the 11.9 ±8.9 mmHg mean IOP decrease experienced by POAG patients who underwent an IOP-lowering by suturelysis after trabeculectomy surgery in our prior study [25]. The average strain response to ICP lowering was in the opposite direction of the strain response to IOP lowering, consistent with the concept of IOP and ICP acting as opposing traction on the LC. Whereas the lowering of IOP made the axial strain $E_{zz}$ of the LC less compressive, leading to a positive DVC calculation for $E_{zz}$ from the higher reference IOP [25], the lowering of ICP made $E_{zz}$ more compressive. Lowering ICP also produced a larger tensile $E_{rr}$ and $E_{\theta \theta }$, and greater $E_{\theta z}$, while lowering IOP did the opposite. Previous studies on animal models also found that IOP and ICP change produced opposing effects on ONH deformation [34,35]. However, the magnitude of the strain response to lowering ICP here differed from that of lowering IOP in Czerpak et al. [25], and the effect was different for different strain components. The average magnitude of the $E_{zz}$ response to ICP change was half that for the same IOP change, while the average $E_{rr}$, $E_{\theta \theta }$, and $E_{\theta z}$ response to ICP lowering were more than twice that for IOP lowering. The average maximum principal strain, $E_{\max}$, and maximum shear strain, $S_{\max}$, in the *RZ* plane for ICP lowering ( $E_{\max}=S_{\max}=1.0\%$) were a half and two thirds, respectively, of those obtained for IOP lowering ( $E_{\max}=1.8\%$, $S_{\max}=1.5\%$) [25]. These findings are consistent with computational modeling studies showing that LC deformation, in general, is more sensitive to IOP than ICP [20–22]. While the LC average strains were small, there were regions of high strains in nearly all eyes. In a preliminary analysis, we averaged $E_{\max}$ over 30 deg sectors of the radial scans, and found that the sector average $E_{\max}$ reached 2–5% for some sectors. These higher local strains may distort the beams and, over time, cause local ischemic injury. Furthermore, computational modeling studies have shown that the elastic mismatch between the stiff extracellular matrix material of the LC beams and the compliant cellular tissues of the LC pores can produce a large strain concentration in the pores [36–39], potentially activating mechanosensitive astrocytes and disrupting axonal transport.

Overall, lowering IOP and ICP altered the strain state of the LC differently. The axial strain $E_{zz}$ dominated the average LC strain response to IOP lowering and was 4.75 times larger in magnitude than the next largest strain component [25]. In contrast, the magnitude of all strain components produced by ICP lowering were comparable to each other, indicating a more complex strain state with a greater presence of in-plane and out-of-plane shear strains. The modeling study of Hua et al. [20] suggested that the ICP can have a greater effect on some aspects of LC deformation. Specifically, the effect of ICP on the rotation of the peripapillary sclera was twice as much as that of IOP. Furthermore, a change in ICP can cause larger retrolamina deformation of the LC than IOP. We also found that the torsional shear strain $E_{\theta z}$ caused by ICP lowering was twice as large and in the opposite direction as produced by IOP lowering in our prior study [25]. However, IOP lowering produced a wide range of motion of the anterior lamina surface (i.e., ALD change) among the different eyes, from −18.20 μm (anterior motion) to 14.14 μm (posterior motion) [25], while ICP lowering in this study produced a smaller range of ALD change from 0.2 to 4.4 μm. However, the mean ALD change was nearly identical for both studies.

All subjects experienced an ICP decrease and a small IOP change, ranging from −2.5 mmHg to 4.1 mmHg, after the extended CSF drainage. The $E_{rr}$, $E_{r\theta }$, $E_{\max}$, and $S_{\max}$ were significantly associated with the individual TLPD change, rather than the separate ICP nor IOP change. This supports the concept that both pressures acting together have an effect on LC strain greater than either alone. However, $E_{zz}$ was significantly associated with the ICP change but not with the TLPD change. ICP lowering may have some effects on LC deformation independent of TLPD, as predicted by modeling studies [20,22].

The study had a number of limitations. First, the sample size was small, which prevented more sophisticated statistical analysis that considered the correlation between two eyes of the same subject. Four of 7 subjects had accurate DVC calculations only for the right eye. For the other 3 subjects with accurate DVC calculations for both eyes, we only included the right eye because the sample size was too small to account for both eyes being associated with the same ICP change. Including both eyes increased the variability of any comparisons involving ICP change. While this did not change the trends of the relationships between strains, ICP change, and TLPD change, the comparisons were not significant (Supplemental Materials on the ASME Digital Collection). The small sample size also precluded multivariate statistical analyses for the effects of age, axial length, baseline IOP, and change in IOP, ICP, and TLPD on the deformation response of the LC. A smaller compressive $E_{zz}$ was associated with older age, and a smaller ALD displacement was associated with a smaller axial length. Accounting for the effects of age and axial length may yield more significant relationships between strains and ICP, IOP, and TLPD change. The baseline IOP varied from 11.8 mmHg to 23.2 mmHg. Zhu et al. [16,18] showed that the effect of ICP and IOP on the morphology of the ONH of monkey eyes depended on their respective levels. The effects of IOP were more dominant for higher ICP, while those of ICP were more dominant for lower IOP. However, the authors examined a far wider range of pressures, 5–45 mmHg for IOP and 5–35 mmHg for ICP. In the narrower IOP range of 11.8 mmHg–23.2 mmHg of this study, ICP and IOP may only interact through the TLPD.

The small sample size also prevented analysis of the regional variations in the LC deformation response. Previously, we showed significant regional relationships between the LC strain response and ALD change that were different from the relationship between the mean ALD change and LC strain response [25]. Preliminary analysis of the strains averaged over 30 deg sectors of the radial scan showed that the sector averaged $E_{\max}$ for some sectors were two to five times higher than the average value for the LC. Similar experiments applying SD-OCT and DVC to monkey and human eyes also have shown local regions with high strains in the LC with ICP change [15,17]. These local regions of high strain may lead to the development of localized axonal damage and tissue remodeling.

The TLPD is defined in this study as the difference between IOP and ICP. However, TLPD would be defined more accurately as the difference between IOP and RLTP, which currently cannot be measured in patients. The RLTP has been estimated from measurements in canine eyes to be about 80% of the ICP for ICP above a threshold of 1.3 mmHg. That study also shows that the RLTP does not fall to zero below the ICP threshold but remains at a minimum of 4 mmHg. However, the scale factor relating RLTP and ICP, threshold ICP, and minimum RLTP measured for dog eyes may not apply to human eyes. We are further aware that the ICP values at the beginning of drainage were taken with the patient in the lateral decubitus position and are, therefore, higher than in the sitting position. Our IOP measurements, however, were taken in the sitting position. The values for change in TLPD are presented with this difference in mind since we have no means to measure ICP at the retrobulbar space, either sitting or lying down.

This study examined the ONH deformation after 2 days of ICP lowering, which was different than the 20-min period of IOP lowering after the suturelysis and the week-long period of IOP lowering with glaucoma medication change. We and others have shown that ONH deformation is time-dependent, exhibiting viscoelastic creep over the span of weeks to months, and slower remodeling over the period of months to years [25,26,33,40–42]. The longer 2-day time period of ICP lowering may lead to greater LC deformation than in previous animal studies that acutely controlled IOP and ICP by cannulation [15,16,18], as well as our previous study lowering IOP by suturelysis.

The study enrolled NPH patients without considering their ophthalmic records. Three of the eyes had a baseline IOP greater than 21 mmHg, and one eye had POAG. The LC of glaucoma eyes with greater structural and functional damage exhibit a more compliant strain response to IOP change [25,26,43]. The LC becomes progressively thinner and more bowed, and consequently, a more compliant structure, with advancing glaucoma, which may alter the effects of ICP change. The effect of glaucoma on the LC strain response should also be considered when comparing the results of this study to those of our previous study of IOP lowering by suturelysis in glaucoma patients [25].

## 5 Conclusions

We applied DVC to SD-OCT scans of the ONH of NPH patients acquired immediately before and after extended CSF drainage to calculate the LC deformation response. The method was able to reliably measure the LC strain response for the range of ICP and IOP changes produced by the extended CSF drainage. The strain response to ICP lowering was in the opposite direction but different in magnitude than the strain response to a similar level of IOP lowering measured in a prior study for POAG patients after suturelysis. In general, the LC strain response to ICP and IOP change occurred as expected in their effect on TLPD. However, the axial strain was significantly associated with ICP change but not with TLPD change. These findings support the hypothesis of ICP being a biomechanical risk factor for glaucoma and motivate the need for a larger study to evaluate the significant effects of ICP change and TLPD change on the LC deformation response.

## Funding Data

- NIH NEI (award ID: R01 036042; Funder ID: 10.13039/100000053).
- NIH NEI R01 02120 (Funder ID: 10.13039/100000053).
- Wilmer Institute Core Grant (award ID: P30 01765; Funder ID: 10.113039100000053).
- Research to Prevent Blindness, the A. Edward Maumenee Professorship (Funder ID: 10.13039/100001818).
- BrightFocus (award ID: G20210125; Funder ID: 10.13039/100006312).

## Data Availability Statement

The datasets generated and supporting the findings of this article are obtainable from the corresponding author upon reasonable request.

## Disclosure

Heidelberg Engineering has provided on loan Spectralis SD-OCT to Dr. Quigley for research studies on ONH biomechanics. However, the present study uses a different Spectralis at the Cerebrospinal Fluid Center, and Heidelberg has not participated in the study design, analyses, or preparation of the paper.

## Supplementary Material

Supplementary Material Supplementary Material Figures

## Appendix: Statistical Analysis of LC Deformation Response

**Table 2:** Linear regression model for the relationship between the ALD change ( μm) and LC strain response to ICP decrease ( n=7)

Row	Intercept ( $c_{0}$)	*p*-val $c_{0}$	ALD $\left(c_{1}\right)$	*p*-val $c_{1}$	$R^{2}$
$E_{zz}$	−6.10 × 10^−3^	1.00 × 10^−1^	6.39 × 10^−4^	6.68 × 10^−1^	3.99 × 10^−2^
$E_{rr}$	7.62 × 10^−3^	4.30 × 10^−2^	−1.36 × 10^−3^	3.47 × 10^−1^	1.77 × 10^−1^
$E_{\theta \theta }$	1.34 × 10^−2^	3.36 × 10^−1^	−1.81 × 10^−3^	7.70 × 10^−1^	1.87 × 10^−2^
$E_{r\theta }$	4.44 × 10^−3^	3.51 × 10^−1^	−1.66 × 10^−3^	4.44 × 10^−1^	1.21 × 10^−1^
$E_{\theta z}$	5.13 × 10^−3^	3.46 × 10^−3^	−1.00 × 10^−3^	7.95 × 10^−2^	4.91 × 10^−1^
$E_{rz}$	5.50 × 10^−4^	5.54 × 10^−1^	−1.40 × 10^−4^	7.41 × 10^−1^	2.38 × 10^−2^
$E_{\max}$	1.27 × 10^−2^	5.63 × 10^−2^	−1.71 × 10^−3^	5.05 × 10^−1^	9.32 × 10^−2^
$S_{\max}$	1.20 × 10^−2^	2.85 × 10^−2^	−1.35 × 10^−3^	4.92 × 10^−1^	9.91 × 10^−2^

Positive ALD change is in the posterior direction.

**Table 3:** Linear regression model for the effect of age (years) on the LC strain and ALD change ( μm) response to ICP decrease ( n=7)

Row	Intercept ( $c_{0}$)	*p*-val $c_{0}$	Age $\left(c_{1}\right)$	*p*-val $c_{1}$	$R^{2}$
$E_{zz}$	−2.61 × 10^−2^	1.20 × 10^−2^	3.23 × 10^−4^	**2.51** × 10^−2^	6.66 × 10^−1^
$E_{rr}$	1.56 × 10^−2^	2.19 × 10^−1^	−1.54 × 10^−4^	3.93 × 10^−1^	1.49 × 10^−1^
$E_{\theta \theta }$	3.32 × 10^−2^	5.21 × 10^−1^	−3.43 × 10^−4^	6.52 × 10^−1^	4.40 × 10^−2^
$E_{r\theta }$	1.28 × 10^−2^	4.87 × 10^−1^	−1.67 × 10^−4^	5.38 × 10^−1^	8.03 × 10^−2^
$E_{\theta z}$	−6.93 × 10^−4^	8.95 × 10^−1^	6.24 × 10^−5^	4.38 × 10^−1^	1.24 × 10^−1^
$E_{rz}$	5.37 × 10^−3^	8.40 × 10^−2^	−7.61 × 10^−5^	9.52 × 10^−2^	4.58 × 10^−1^
$E_{\max}$	2.53 × 10^−2^	2.54 × 10^−1^	−2.33 × 10^−3^	4.61 × 10^−1^	1.13 × 10^−1^
$S_{\max}$	3.08 × 10^−2^	6.12 × 10^−2^	−3.18 × 10^−4^	1.56 × 10^−1^	3.58 × 10^−1^
ALD	2.25 × 10	5.71 × 10^−1^	8.71 × 10^−3^	8.81 × 10^−1^	4.95 × 10^−3^

Positive ALD change is in the posterior direction.

**Table 4:** Linear regression model for the effect of axial length (mm) on the LC strain and ALD change ( μm) response to ICP decrease ( n=7)

Row	Intercept ( $c_{0}$)	*p*-val $c_{0}$	Axial Length $\left(c_{1}\right)$	*p*-val $c_{1}$	$R^{2}$
$E_{zz}$	−1.25 × 10^−2^	7.63 × 10^−1^	3.11 × 10^−4^	8.57 × 10^−1^	7.18 × 10^−3^
$E_{rr}$	3.30 × 10^−2^	4.21 × 10^−1^	−1.16 × 10^−3^	4.95 × 10^−1^	9.76 × 10^−2^
$E_{\theta \theta }$	1.36 × 10^−1^	4.13 × 10^−1^	−5.25 × 10^−3^	4.47 × 10^−1^	1.20 × 10^−1^
$E_{r\theta }$	4.71 × 10^−2^	4.32 × 10^−1^	−1.90 × 10^−3^	4.47 × 10^−1^	1.20 × 10^−1^
$E_{\theta z}$	2.13 × 10^−2^	2.31 × 10^−1^	−7.47 × 10^−4^	3.05 × 10^−1^	2.07 × 10^−1^
$E_{rz}$	2.43 × 10^−3^	8.36 × 10^−1^	−8.82 × 10^−5^	8.57 × 10^−1^	7.14 × 10^−3^
$E_{\max}$	5.53 × 10^−2^	4.40 × 10^−1^	−1.90 × 10^−3^	5.21 × 10^−1^	8.69 × 10^−2^
$S_{\max}$	4.50 × 10^−2^	4.13 × 10^−1^	−1.47 × 10^−3^	5.21 × 10^−1^	8.95 × 10^−2^
ALD	−2.05 × 10^1^	3.70 × 10^−2^	9.28 × 10^−1^	**2.82** × 10^−2^	6.51 × 10^−1^

Positive ALD change is in the posterior direction.

Bold values indicate stastistically significant comparisons, where *p* < 0.05.

**Table 5:** Linear regression model for the effect of baseline IOP (mmHg) on the LC strain and ALD change ( μm) response to ICP decrease ( n=7)

Row	Intercept ( $c_{0}$)	*p*-val $c_{0}$	Baseline IOP $\left(c_{1}\right)$	*p*-val $c_{1}$	$R^{2}$
$E_{zz}$	1.67 × 10^−3^	8.21 × 10^−1^	−3.78 × 10^−4^	3.68 × 10^−1^	1.64 × 10^−1^
$E_{rr}$	−8.86 × 10^−4^	9.06 × 10^−1^	3.52 × 10^−4^	4.09 × 10^−1^	1.39 × 10^−1^
$E_{\theta \theta }$	−1.78 × 10^−2^	5.63 × 10^−1^	1.60 × 10^−3^	3.57 × 10^−1^	1.71 × 10^−1^
$E_{r\theta }$	−4.69 × 10^−3^	6.88 × 10^−1^	3.59 × 10^−4^	5.77 × 10^−1^	6.63 × 10^−2^
$E_{\theta z}$	3.84 × 10^−3^	3.12 × 10^−1^	−2.20 × 10^−5^	9.11 × 10^−1^	2.78 × 10^−3^
$E_{rz}$	−1.33 × 10^−3^	5.44 × 10^−1^	9.27 × 10^−5^	4.43 × 10^−1^	1.22 × 10^−1^
$E_{\max}$	−9.09 × 10^−4^	9.45 × 10^−1^	6.09 × 10^−4^	4.11 × 10^−1^	1.39 × 10^−1^
$S_{\max}$	−1.30 × 10^−3^	8.89 × 10^−1^	6.22 × 10^−4^	2.57 × 10^−1^	2.47 × 10^−1^
ALD	2.88 × 10	2.72 × 10^−1^	−6.80 × 10^−2^	6.16 × 10^−1^	5.41 × 10^−2^

Positive ALD change is in the posterior direction.

**Table 6:** Linear regression model for the effect of ICP change (mmHg) on the LC strain and ALD change ( μm) response ( n=7)

Row	Intercept ( $c_{0}$)	*p*-val $c_{0}$	ICP Change $\left(c_{1}\right)$	*p*-val $c_{1}$	$R^{2}$
$E_{zz}$	2.08 × 10^−2^	1.72 × 10^−2^	2.30 × 10^−3^	**6.99** × 10^−3^	7.95 × 10^−1^
$E_{rr}$	−6.75 × 10^−3^	5.98 × 10^−1^	−1.08 × 10^−3^	3.55 × 10^−1^	1.72 × 10^−1^
$E_{\theta \theta }$	−2.21 × 10^−2^	6.89 × 10^−1^	−2.89 × 10^−3^	5.55 × 10^−1^	7.40 × 10^−1^
$E_{r\theta }$	−2.16 × 10^−2^	2.45 × 10^−1^	−2.07 × 10^−3^	2.11 × 10^−1^	2.91 × 10^−3^
$E_{\theta z}$	4.48 × 10^−3^	4.77 × 10^−1^	9.14 × 10^−5^	8.65 × 10^−1^	6.32 × 10^−2^
$E_{rz}$	−4.74 × 10^−3^	1.68 × 10^−1^	−4.49 × 10^−4^	1.42 × 10^−1^	3.77 × 10^−1^
$E_{\max}$	−7.67 × 10^−3^	7.36 × 10^−1^	−1.56 × 10^−3^	4.47 × 10^−1^	1.20 × 10^−1^
$S_{\max}$	−1.47 × 10^−2^	3.31 × 10^−1^	−2.17 × 10^−3^	1.30 × 10^−1^	3.96 × 10^−1^
ALD	6.05 × 10	1.52 × 10^−1^	3.89 × 10^−1^	2.72 × 10^−1^	2.34 × 10^−1^

Positive ALD change is in the posterior direction.

Bold values indicate stastistically significant comparisons, where *p* < 0.05.

**Table 7:** Linear regression model for the effect of IOP change (mmHg) on the LC strain and ALD change ( μm) response ( n=7)

Row	Intercept ( $c_{0}$)	*p*-val $c_{0}$	IOP change $\left(c_{1}\right)$	*p*-val $c_{1}$	$R^{2}$
$E_{zz}$	−5.19 × 10^−3^	4.99 × 10^−2^	2.41 × 10^−4^	7.81 × 10^−1^	1.69 × 10^−2^
$E_{rr}$	4.43 × 10^−3^	2.45 × 10^−2^	1.37 × 10^−3^	6.00 × 10^−2^	5.40 × 10^−1^
$E_{\theta \theta }$	7.98 × 10^−3^	3.29 × 10^−1^	3.64 × 10^−3^	2.80 × 10^−1^	2.27 × 10^−1^
$E_{r\theta }$	1.90 × 10^−3^	5.55 × 10^−1^	−3.55 × 10^−4^	7.83 × 10^−1^	1.66 × 10^−2^
$E_{\theta z}$	3.30 × 10^−3^	1.30 × 10^−2^	2.26 × 10^−4^	5.53 × 10^−1^	7.46 × 10^−2^
$E_{rz}$	1.12 × 10^−4^	8.22 × 10^−1^	3.03 × 10^−4^	1.74 × 10^−1^	3.34 × 10^−1^
$E_{\max}$	8.32 × 10^−3^	1.99 × 10^−2^	2.33 × 10^−3^	6.76 × 10^−2^	5.20 × 10^−1^
$S_{\max}$	8.70 × 10^−3^	9.83 × 10^−3^	1.53 × 10^−3^	1.40 × 10^−1^	3.80 × 10^−1^
ALD	1.66 × 10	4.79 × 10^−2^	2.36 × 10^−2^	9.31 × 10^−1^	1.66 × 10^−3^

Positive ALD change is in the posterior direction.

**Table 8:** Linear regression model for the effect of TLPD (IOP-ICP) change (mmHg) on the LC strain and ALD change ( μm) response ( n=7)

Row	Intercept ( $c_{0}$)	*p*-val $c_{0}$	TLPD Change $\left(c_{1}\right)$	*p*-val $c_{1}$	$R^{2}$
$E_{zz}$	2.40 × 10^−3^	7.62 × 10^−1^	−6.23 × 10^−4^	3.55 × 10^−1^	1.72 × 10^−1^
$E_{rr}$	−9.8 × 10^−3^	8.25 × 10^−2^	1.27 × 10^−3^	**1.83** × 10^−2^	7.04 × 10^−1^
$E_{\theta \theta }$	−2.99 × 10^−2^	3.42 × 10^−1^	3.39 × 10^−3^	2.05 × 10^−1^	2.98 × 10^−1^
$E_{r\theta }$	−3.95 × 10^−3^	7.56 × 10^−1^	4.71 × 10^−4^	6.51 × 10^−1^	4.42 × 10^−2^
$E_{\theta z}$	2.05 × 10^−3^	5.97 × 10^−1^	1.18 × 10^−4^	7.07 × 10^−1^	3.08 × 10^−2^
$E_{rz}$	−3.90 × 10^−3^	3.13 × 10^−2^	3.53 × 10^−4^	**2.14** × 10^−2^	6.86 × 10^−1^
$E_{\max}$	−1.48 × 10^−2^	1.56 × 10^−1^	2.07 × 10^−3^	**3.53** × 10^−2^	6.21 × 10^−1^
$S_{\max}$	−1.11 × 10^−2^	9.84 × 10^−2^	1.75 × 10^−3^	**1.12** × 10^−2^	7.54 × 10^−1^
ALD	3.07 × 10	2.74 × 10^−1^	−1.17 × 10^−1^	5.90 × 10^−1^	6.21 × 10^−2^

Positive ALD change is in the posterior direction.

Bold values indicate stastistically significant comparisons, where *p* < 0.05.

## References

[1] Morgan, W. H., Yu, D. Y., Alder, V. A., Cringle, S. J., Cooper, R. L., House, P. H., and Constable, I. J., 1998, “The Correlation Between Cerebrospinal Fluid Pressure and Retrolaminar Tissue Pressure,” Investig. Ophthalmol. Visual Sci., 39(8), pp. 1419–1428.https://iovs.arvojournals.org/article.aspx?articleid=2161891 9660490

[2] Berdahl, J. P., and Allingham, R. R., 2010, “Intracranial Pressure and Glaucoma,” Curr. Opin. Ophthalmol., 21(2), pp. 106–111. 10.1097/ICU.0b013e32833651d8 20040876

[3] Chowdhury, U. R., and Fautsch, M. P., 2015, “Intracranial Pressure and Its Relationship to Glaucoma: Current Understanding and Future Directions,” Med. Hypothesis, Discovery Innovation. Ophthalmol. J., 4(3), pp. 71–80.https://pubmed.ncbi.nlm.nih.gov/27350948/PMC4921207 27350948

[4] Berdahl, J. P., Allingham, R. R., and Johnson, D. H., 2008, “Cerebrospinal Fluid Pressure Is Decreased in Primary Open-Angle Glaucoma,” Ophthalmology, 115(5), pp. 763–768. 10.1016/j.ophtha.2008.01.013 18452762

[5] Berdahl, J. P., Fautsch, M. P., Stinnett, S. S., and Allingham, R. R., 2008, “Intracranial Pressure in Primary Open Angle Glaucoma, Normal Tension Glaucoma, and Ocular Hypertension: A Case-Control Study,” Invest. Ophthalmol. Visual Sci., 49(12), pp. 5412–5418. 10.1167/iovs.08-2228 18719086 PMC2745832

[6] Ren, R., Jonas, J. B., Tian, G., Zhen, Y., Ma, K., Li, S., Wang, H., Li, B., Zhang, X., and Wang, N., 2010, “Cerebrospinal Fluid Pressure in Glaucoma: A Prospective Study,” Ophthalmology, 117(2), pp. 259–266. 10.1016/j.ophtha.2009.06.058 19969367

[7] Siaudvytyte, L., Januleviciene, I., Ragauskas, A., Bartusis, L., Meiliuniene, I., Siesky, B., and Harris, A., 2014, “The Difference in Translaminar Pressure Gradient and Neuroretinal Rim Area in Glaucoma and Healthy Subjects,” J. Ophthalmol., 2014, pp. 1–5. 10.1155/2014/937360 PMC4021754 24876948

[8] Pircher, A., Remonda, L., Weinreb, R. N., and Killer, H. E., 2017, “Translaminar Pressure in Caucasian Normal Tension Glaucoma Patients,” Acta Ophthalmol., 95(7), pp. e524–e531. 10.1111/aos.13302 27966838

[9] Lindén, C., Qvarlander, S., Jóhannesson, G., Johansson, E., Östlund, F., Malm, J., and Eklund, A., 2018, “Normal-Tension Glaucoma Has Normal Intracranial Pressure,” Ophthalmology, 125(3), pp. 361–368. 10.1016/j.ophtha.2017.09.022 29096996

[10] Rudolph, D., Sterker, I., Graefe, G., Till, H., Ulrich, A., and Geyer, C., 2010, “Visual Field Constriction in Children With Shunt-Treated Hydrocephalus,” J. Neurosurg. Pediatr., 6(5), pp. 481–485. 10.3171/2010.8.PEDS1042 21039173

[11] Gallina, P., Savastano, A., Becattini, E., Orlandini, S., Scollato, A., Rizzo, S., Carreras, G., Di Lorenzo, N., and Porfirio, B., 2018, “Glaucoma in Patients With Shunt-Treated Normal Pressure Hydrocephalus,” J. Neurosurg., 129(4), pp. 1078–1084. 10.3171/2017.5.JNS163062 29148901

[12] Yang, D., Fu, J., Hou, R., Liu, K., Jonas, J. B., Wang, H., Chen, W., ., 2014, “Optic Neuropathy Induced by Experimentally Reduced Cerebrospinal Fluid Pressure in Monkeys,” Invest. Ophthalmol. Visual Sci., 55(5), pp. 3067–3073. 10.1167/iovs.13-13657 24736050

[13] Lee, D. S., Lee, E. J., Kim, T.-W., Park, Y. H., Kim, J., Lee, J. W., and Kim, S., 2015, “Influence of Translaminar Pressure Dynamics on the Position of the Anterior Lamina Cribrosa Surface,” Investig. Opthalmology Visual Sci., 56(5), p. 2833. 10.1167/iovs.14-15869 25829411

[14] Wang, B., Sigal, I. A., Smith, M. A., Kostanyan, T., Bilonick, R. A., Tran, H., Kagemanm, L., Tyler-Kabara, E., Schuman, J. S., and Wollstein, G., 2015, “In-Vivo 3D Deformation of Lamina Cribrosa Microstructure in Response to Acute Changes in Intraocular and Cerebrospinal Fluid Pressures,” Invest. Ophthalmol. Vis. Sci. 56(7), p. 3979.https://iovs.arvojournals.org/article.aspx?articleid=2333886

[15] Tran, H., Grimm, J., Wang, B., Smith, M. A., Gogola, A., Nelson, S., Tyler-Kabara, E., Schuman, J., Wollstein, G., and Sigal, I. A., 2017, “Mapping in-Vivo Optic Nerve Head Strains Caused by Intraocular and Intracranial Pressures,” Proc. SPIE Int. Soc. Opt. Eng. **10067**, p. 100670B. 10.1117/12.2257360 PMC5880553 29618852

[16] Zhu, Z., Waxman, S., Wang, B., Wallace, J., Schmitt, S. E., Tyler-Kabara, E., Ishikawa, H., ., 2021, “Interplay Between Intraocular and Intracranial Pressure Effects on the Optic Nerve Head in Vivo,” Exp. Eye Res., 213, p. 108809. 10.1016/j.exer.2021.108809 34736887 PMC8665145

[17] Fazio, M. A., Clark, M. E., Bruno, L., and Girkin, C. A., 2018, “In Vivo Optic Nerve Head Mechanical Response to Intraocular and Cerebrospinal Fluid Pressure: Imaging Protocol and Quantification Method,” Sci. Rep., 8(1), p. 12639. 10.1038/s41598-018-31052-x 30140057 PMC6107503

[18] Zhu, Z., Waxman, S., Wang, B., Wallace, J., Schmitt, S. E., Tyler-Kabara, E., Ishikawa, H., ., 2023, “In Vivo Modulation of Intraocular and Intracranial Pressures Causes Nonlinear and Non-monotonic Deformations of the Lamina Cribrosa and Scleral Canal,” bioRxiv. 10.1101/2023.01.29.526113

[19] Feola, A. J., Myers, J. G., Raykin, J., Mulugeta, L., Nelson, E. S., Samuels, B. C., and Ethier, C. R., 2016, “Finite Element Modeling of Factors Influencing Optic Nerve Head Deformation Due to Intracranial Pressure,” Investig. Opthalmol. Visual Sci., 57(4), p. 1901. 10.1167/iovs.15-17573 27088762

[20] Hua, Y., Voorhees, A. P., and Sigal, I. A., 2018, “Cerebrospinal Fluid Pressure: Revisiting Factors Influencing Optic Nerve Head Biomechanics,” Invest. Ophthalmol. Visual Sci., 59(1), pp. 154–165. 10.1167/iovs.17-22488 29332130 PMC5769499

[21] Tong, J., Ghate, D., Kedar, S., and Gu, L., 2019, “Relative Contributions of Intracranial Pressure and Intraocular Pressure on Lamina Cribrosa Behavior,” J. Ophthalmol., 2019, p. 3064949. 10.1155/2019/3064949 31007950 PMC6441528

[22] Karimi, A., Rahmati, S. M., Grytz, R. G., Girkin, C. A., and Downs, J. C., 2021, “Modeling the Biomechanics of the Lamina Cribrosa Microstructure in the Human Eye,” Acta Biomater., 134, pp. 357–378. 10.1016/j.actbio.2021.07.010 34245889 PMC8542639

[23] Hua, Y., Tong, J., Ghate, D., Kedar, S., and Gu, L., 2016, “Intracranial Pressure Influences the Behavior of Optic Nerve Head,” ASME J. Biomech. Eng., 139(3), p. 031003. 10.1115/1.4035406 27935009

[24] Midgett, D. E. D. E., Quigley, H. A. H. A., and Nguyen, T. D. T. D., 2019, “In Vivo Characterization of the Deformation of the Human Optic Nerve Head Using Optical Coherence Tomography and Digital Volume Correlation,” Acta Biomater., 96, pp. 385–399. 10.1016/j.actbio.2019.06.050 31279161 PMC6717668

[25] Czerpak, C. A., Ling, Y. T. T., Jefferys, J. L., Quigley, H. A., and Nguyen, T. D., 2023, “The Curvature, Collagen Network Structure, and Their Relationship to the Pressure-Induced Strain Response of the Human Lamina Cribrosa in Normal and Glaucoma Eyes,” ASME J. Biomech. Eng., 145(10), p. 101005. 10.1115/1.4062846 PMC10405282 37382629

[26] Hannay, V., 2023, “In Vivo Biomechanical Strain Response of the Lamina Cribrosa to Pressure Change as a Result of Glaucoma Medication Change,” Ph.D. thesis, Johns Hopkins University, Baltimore, MD. 10.1016/j.xops.2024.100473

[27] Haan, J., and Thomeer, R. T., 1988, “Predictive Value of Temporary External Lumbar Drainage in Normal Pressure Hydrocephalus,” Neurosurgery, 22(2), pp. 388–391. 10.1227/00006123-198802000-00020 3352890

[28] Toma, A. K., 2023, “Extended Lumbar Drainage: Supplementary Test to Diagnose Shunt Responsive iNPH,” Normal Pressure Hydrocephalus, Springer International Publishing, Cham, pp. 209–219.

[29] Schindelin, J., Arganda-Carreras, I., Frise, E., Kaynig, V., Longair, M., Pietzsch, T., Preibisch, S., ., 2012, “Fiji: An Open-Source Platform for Biological-Image Analysis,” Nat. Methods, 9(7), pp. 676–682. 10.1038/nmeth.2019 22743772 PMC3855844

[30] Clingo, K. A., Czerpak, C. A., Quigley, H. A., and Nguyen, T. D., 2024, “Semi-Automated Segmentation of ONH Tissues Using Deep Learning,” Invest. Ophthalmol. Visual Sci., 65, p. 2492.https://iovs.arvojournals.org/article.aspx?articleid=2795000

[31] Bar-Kochba, E., Toyjanova, J., Andrews, E., Kim, K.-S., and Franck, C., 2015, “A Fast Iterative Digital Volume Correlation Algorithm for Large Deformations,” Exp. Mech., 55(1), pp. 261–274. 10.1007/s11340-014-9874-2

[32] Czerpak, C. A., Kashaf, M. S., Zimmerman, B. K., Quigley, H. A., and Nguyen, T. D., 2023, “The Strain Response to Intraocular Pressure Decrease in the Lamina Cribrosa of Patients With Glaucoma,” Ophthalmol. Glaucoma, 6(1), pp. 11–22. 10.1016/j.ogla.2022.07.005 35863747 PMC9849479

[33] Czerpak, C. A., Quigley, H. A., and Nguyen, T. D., 2024, “Long-Term Remodeling Response in the Lamina Cribrosa Years After Intraocular Pressure Lowering by Suturelysis After Trabeculectomy,” Ophthalmol. Glaucoma, 7(3), pp. 298–307. 10.1016/j.ogla.2024.01.003 38272391 PMC11127792

[34] Morgan, W. H., Chauhan, B. C., Yu, D. Y., Cringle, S., Alder, V. A., and House, P. H., 2002, “Optic Disc Movement With Variations in Intraocular and Cerebrospinal Fluid Pressure,” Invest. Ophthalmol. Visual Sci., 43(10), pp. 3236–3242.https://iovs.arvojournals.org/article.aspx?articleid=2122964 12356830

[35] Zhao, D., He, Z., Vingrys, A. J., Bui, B. V., and Nguyen, C. T. O., 2015, “The Effect of Intraocular and Intracranial Pressure on Retinal Structure and Function in Rats,” Physiol. Rep., 3(8), p. e12507. 10.14814/phy2.12507 26290528 PMC4562590

[36] Ling, Y. T. T., Korneva, A., Quigley, H. A., and Nguyen, T. D., 2023, “Computational Study of the Mechanical Behavior of the Astrocyte Network and Axonal Compartments in the Mouse Optic Nerve Head,” Biomech. Model. Mechanobiol., 22(5), pp. 1751–1772. 10.1007/s10237-023-01752-z 37573553 PMC10988382

[37] Voorhees, A. P., Jan, N. J., and Sigal, I. A., 2017, “Effects of Collagen Microstructure and Material Properties on the Deformation of the Neural Tissues of the Lamina Cribrosa,” Acta Biomater., 58, pp. 278–290. 10.1016/j.actbio.2017.05.042 28528864 PMC5537032

[38] Roberts, M. D., Liang, Y., Sigal, I. A., Grimm, J., Reynaud, J., Bellezza, A., Burgoyne, C. F., and Downs, J. C., 2010, “Correlation Between Local Stress and Strain and Lamina Cribrosa Connective Tissue Volume Fraction in Normal Monkey Eyes,” Invest. Opthalmol. Visual Sci., 51(1), p. 295. 10.1167/iovs.09-4016 PMC2829275 19696175

[39] Downs, J. C., Roberts, M. D., Burgoyne, C. F., and Hart, R. T., 2009, “Multiscale Finite Element Modeling of the Lamina Cribrosa Microarchitecture in the Eye,” Annual International Conference of the IEEE Engineering in Medicine and Biology Society, IEEE, Minneapolis, MN, Sept. 3--6, pp. 4277–4280. 10.1109/IEMBS.2009.5332755 PMC3268703 19963817

[40] Kadziauskienė, A., Jašinskienė, E., Ašoklis, R., Lesinskas, E., Rekašius, T., Chua, J., Cheng, C.-Y., Mari, J. M., Girard, M. J. A., and Schmetterer, L., 2018, “Long-Term Shape, Curvature, and Depth Changes of the Lamina Cribrosa After Trabeculectomy,” Ophthalmology, 125(11), pp. 1729–1740. 10.1016/j.ophtha.2018.05.011 29961552

[41] Krzyżanowska-Berkowska, P., Czajor, K., Helemejko, I., and Iskander, D. R., 2018, “Relationship Between the Rate of Change in Lamina Cribrosa Depth and the Rate of Retinal Nerve Fiber Layer Thinning Following Glaucoma Surgery,” PLoS One, 13(11), p. e0206040. 10.1371/journal.pone.0206040 30399148 PMC6219770

[42] Krzyżanowska-Berkowska, P., Melińska, A., Helemejko, I., and Iskander, D. R., 2018, “Evaluating Displacement of Lamina Cribrosa Following Glaucoma Surgery. Graefe's Arch,” Clin. Exp. Ophthalmol., 256(4), pp. 791–800. 10.1007/s00417-018-3920-1 PMC5856897 29423838

[43] Chuangsuwanich, T., Tun, T. A., Braeu, F. A., Wang, X., Chin, Z. Y., Panda, S. K., Buist, M., ., 2023, “Differing Associations Between Optic Nerve Head Strains and Visual Field Loss in Patients With Normal- and High-Tension Glaucoma,” Ophthalmology, 130(1), pp. 99–110. 10.1016/j.ophtha.2022.08.007 35964710

