# Supplementary material for: Deformation Response of the Human Lamina Cribrosa to Intracranial Pressure Lowering
Source: J Biomech Eng. 2025 Jun 4;147(7):071008. doi: 10.1115/1.4068633 (PMC12502050; doi:10.1115/1.4068633)
Supplement: Supplementary Material — Figures [file Supplementary_Material_BIO-24-1320.zip › Supplementary_Material_BIO-24-1320.pdf]

Supplemental Information

May 4, 2025

This Supplemental Information presents the strain data and statistical analysis for 10 eyes of 7 patients. LC strains could be calculated only for the right eye

1 The strain response and ALD change

Table S1: The DVC calculated strains and baseline strain errors for $n = 10$ eyes of 7 patients.

Strains	Response to ICP		p-val	Baseline Error		p-val
	Mean	Std	strain $\neq 0$	Mean	Std	strain $\neq$error
E_{zz}	-4.48E-03	4.16E-03	**0.0078**	9.36E-04	9.97E-04	**0.0039**
E_{rr}	3.44E-03	4.96E-03	0.0559	1.56E-05	8.81E-04	0.0591
$E_{\theta\theta}$	7.02E-03	1.69E-02	0.22	-2.396E-04	9.21E-04	0.20
$E_{r\theta}$	2.61E-03	6.27E-03	0.22	-5.94E-05	9.07E-04	0.18
$E_{\theta z}$	3.33E-03	1.98E-03	**0.00048**	1.17E-04	8.28E-03	0.21
E_{rz}	5.70E-04	1.38E-03	0.22	1.63E-04	5.77E-04	0.38

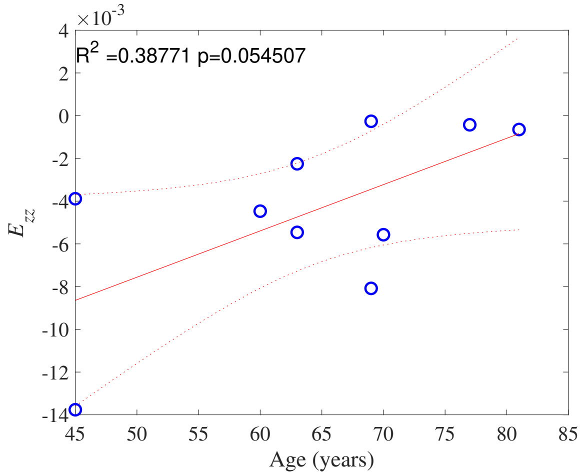

Figure S1: The compressive E_{zz} response is stiffer with older age ($p = 0.054$, $n = 10$ eyes of 7 patients).

ICP lowering produced a small nonsignificant ALD change (mean: 1.10 ± 1.73 μm, $p = 0.0737$, $n = 10$ eyes of 7 patients).

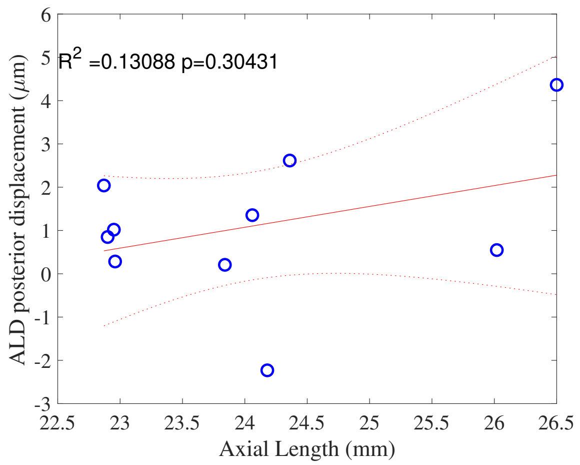

Figure S2: ALD change was not associated with greater axial length ($n = 10$ eyes of 7 patients).

2 Relationships between the LC strain response, ICP, IOP, and TLPD

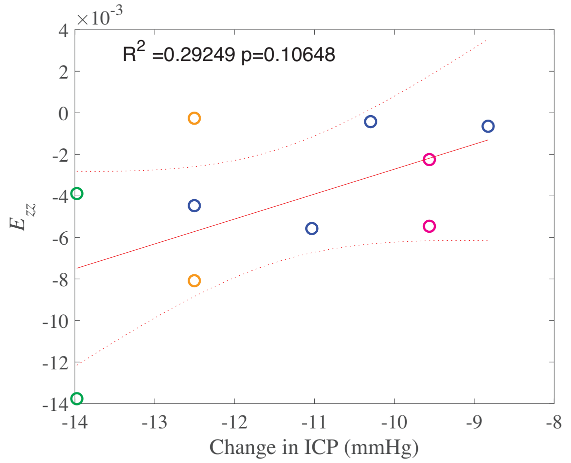

Figure S3: E_{zz} was not associated with ICP decrease ($n = 10$ eyes of 7 patients). The blue circles are strain data for the 4 right eyes of 4 patients, and the green, orange, and magenta circles are strain data for the left and right eyes of 3 patients. There was a large difference in the average E_{zz} response of the LC between the left and right eyes, and associating both with the same ICP measured for the patient led to a large scatter in the data.

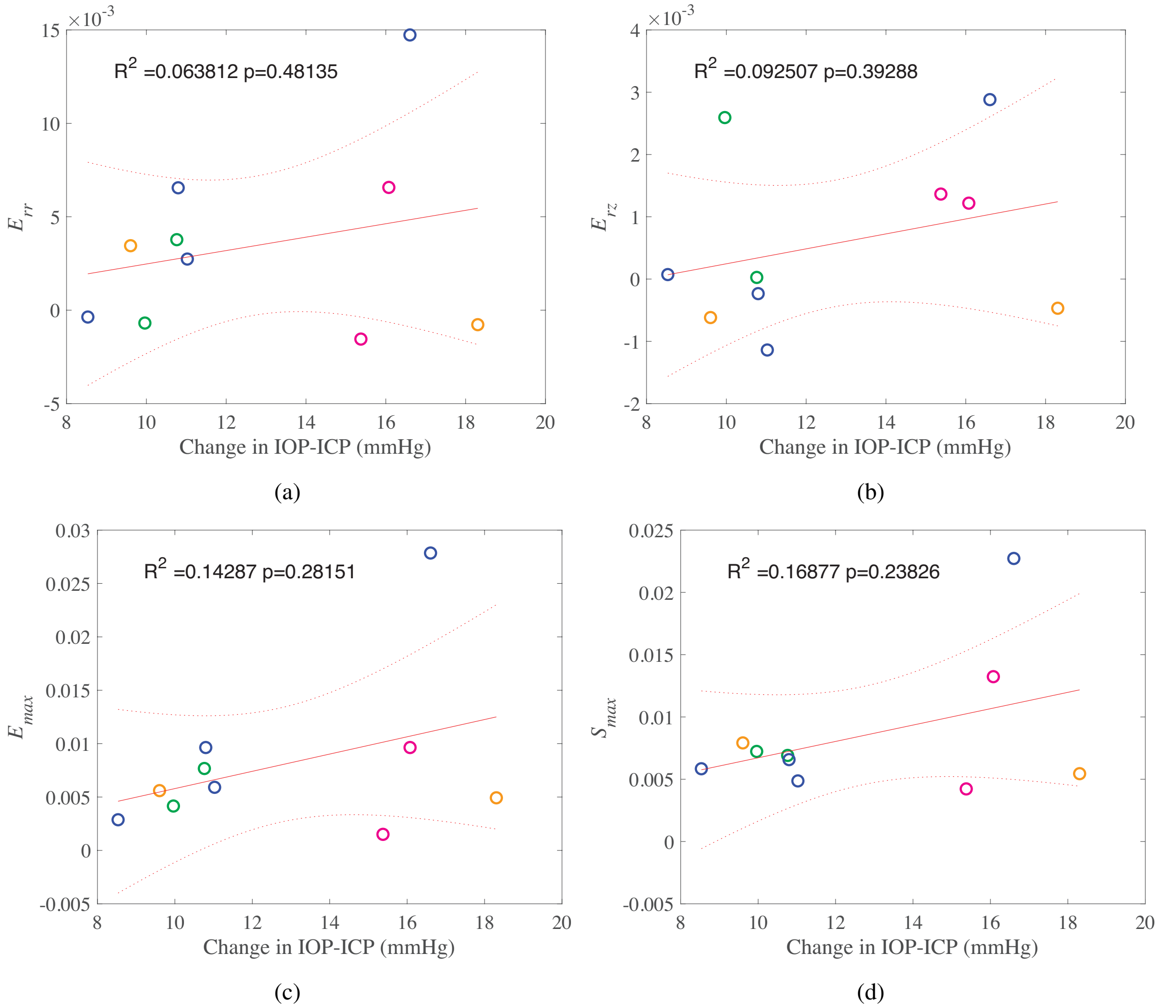

Figure S4: The strain response was not associated with TLPD (IOP-ICP) increase ($n = 10$ eyes of 7 patients): (a) E_{rr}, (b) E_{rz}, (c) E_{max}, and (d) S_{max}. The blue circles are strain data for the 4 right eyes of 4 patients and the green, orange, and magenta circles are strain data of the left and right eyes of 3 patients. There was a large variation in the average E_{zz} response of the LC between the left and right eyes. For 2 of 3 patients, the TLPD change was similar for both eyes because the IOP changes in both eyes were small, and the TLPD change was dominated by the ICP change.

Table S2: Linear regression model for the relationship between the ALD change (μm) and LC strain response to ICP decrease ($n = 10$ eyes of 7 patients). Positive ALD change is in the posterior direction.

Row	Intercept (c_0)	p-val c_0	ALD (c_1)	p-val c_1	R^2
E_{zz}	-4.664E-03	2.394E-02	1.643E-04	8.515E-01	4.655E-03
E_{rr}	3.071E-03	1.622E-01	3.378E-04	7.464E-01	1.381E-02
$E_{\theta\theta}$	6.368E-03	3.779E-01	5.886E-04	8.689E-01	3.619E-03
$E_{r\theta}$	3.577E-03	1.844E-01	-8.719E-04	5.041E-01	5.764E-02
$E_{\theta z}$	3.798E-03	9.371E-04	-4.195E-04	2.985E-01	1.338E-01
E_{rz}	8.391E-04	1.526E-01	-2.438E-04	3.906E-01	9.336E-02
E_{max}	7.602E-03	3.566E-02	3.402E-04	8.291E-01	6.180E-03
S_{max}	8.399E-03	5.889E-03	8.915E-05	9.398E-01	7.594E-04

Table S3: Linear regression model for the effect of Age (years) on the LC strain and ALD change (μm) response to ICP decrease ($n = 10$ eyes of 7 patients). Positive ALD change is in the posterior direction.

Row	Intercept (c_0)	p-val c_0	Age (c_1)	p-val c_1	R^2
E_{zz}	-1.840E-02	1.898E-02	2.167E-04	5.451E-02	3.877E-01
E_{rr}	4.518E-03	6.495E-01	-1.673E-05	9.121E-01	1.620E-03
$E_{\theta\theta}$	1.505E-02	6.553E-01	-1.252E-04	8.080E-01	7.829E-03
$E_{r\theta}$	7.998E-03	5.220E-01	-8.387E-05	6.593E-01	2.552E-02
$E_{\theta z}$	5.154E-04	8.922E-01	4.391E-05	4.597E-01	7.013E-02
E_{rz}	5.159E-03	3.859E-02	-7.149E-05	5.604E-02	3.840E-01
E_{max}	1.074E-02	4.766E-01	-4.307E-05	8.501E-01	4.740E-03
S_{max}	1.768E-02	1.232E-01	-1.431E-04	3.900E-01	9.359E-02
ALD	-2.181E+00	5.038E-01	5.118E-02	3.155E-01	1.253E-01

Table S4: Linear regression model for the effect of Axial Length (mm) on the LC strain and ALD change (μm) response to ICP decrease ($n = 10$ eyes of 7 patients). Positive ALD change is in the posterior direction.

Row	Intercept (c_0)	p-val c_0	Axial Length (c_1)	p-val c_1	R^2
E_{zz}	1.641E-04	9.953E-01	-1.931E-04	8.686E-01	3.632E-03
E_{rr}	3.091E-02	3.494E-01	-1.141E-03	4.024E-01	8.902E-02
$E_{\theta\theta}$	9.978E-02	3.738E-01	-3.855E-03	4.062E-01	8.767E-02
$E_{r\theta}$	4.151E-02	3.159E-01	-1.616E-03	3.449E-01	1.119E-01
$E_{\theta z}$	5.817E-03	6.657E-01	-1.032E-04	8.528E-01	4.570E-03
E_{rz}	-6.694E-03	4.625E-01	3.018E-04	4.261E-01	8.079E-02
E_{max}	4.848E-02	3.316E-01	-1.683E-03	4.125E-01	8.545E-02
S_{max}	3.295E-02	3.823E-01	-1.016E-03	5.115E-01	5.571E-02
ALD	-1.048E+01	3.504E-01	4.814E-01	3.043E-01	1.309E-01

Table S5: Linear regression model for the effect of baseline IOP (mmHg) on the LC strain and ALD change (μm) response to ICP decrease ($n = 10$ eyes of 7 patients). Positive ALD change is in the posterior direction.

Row	Intercept (c_0)	p-val c_0	Baseline IOP (c_1)	p-val c_1	R^2
E_{zz}	-5.076E-04	9.242E-01	-2.197E-04	4.488E-01	7.344E-02
E_{rr}	1.035E-03	8.746E-01	1.332E-04	7.047E-01	1.892E-02
$E_{\theta\theta}$	-1.032E-02	6.344E-01	9.585E-04	4.148E-01	8.463E-02
$E_{r\theta}$	-4.899E-04	9.528E-01	1.716E-04	6.991E-01	1.968E-02
$E_{\theta z}$	4.513E-03	1.112E-01	-6.517E-05	6.412E-01	2.848E-02
E_{rz}	-4.663E-04	7.954E-01	5.727E-05	5.544E-01	4.542E-02
E_{max}	3.633E-03	7.126E-01	2.402E-04	6.491E-01	2.716E-02
S_{max}	3.370E-03	6.416E-01	2.834E-04	4.678E-01	6.770E-02
ALD	3.846E+00	8.959E-02	-1.515E-01	1.920E-01	2.025E-01

Table S6: Linear regression model for the effect of ICP change (mmHg) on the LC strain and ALD change (μm) response ($n = 10$ eyes of 7 patients). Positive ALD change is in the posterior direction.

Row	Intercept (c_0)	p-val c_0	ICP Change (c_1)	p-val c_1	R^2
E_{zz}	9.286E-03	2.601E-01	1.200E-03	1.065E-01	2.925E-01
E_{rr}	-1.116E-04	9.920E-01	-3.099E-04	7.476E-01	1.368E-02
$E_{\theta\theta}$	-7.504E-03	8.429E-01	-1.266E-03	6.989E-01	1.971E-02
$E_{r\theta}$	-1.446E-02	2.736E-01	-1.488E-03	1.978E-01	1.978E-01
$E_{\theta z}$	6.812E-03	1.398E-01	3.031E-04	4.217E-01	8.225E-02
E_{rz}	-1.812E-03	5.490E-01	-2.076E-04	4.295E-01	7.967E-02
E_{max}	1.981E-03	9.058E-01	-5.226E-04	7.182E-01	1.718E-02
S_{max}	-2.606E-03	8.275E-01	-9.676E-04	3.601E-01	1.054E-01
ALD	6.976E+00	5.740E-02	5.117E-01	9.563E-02	3.084E-01

Table S7: Linear regression model for the effect of IOP change (mmHg) on the LC strain and ALD change (μm) response ($n = 10$ eyes of 7 patients). Positive ALD change is in the posterior direction.

Row	Intercept (c_0)	p-val c_0	IOP Change (c_1)	p-val c_1	R^2
E_{zz}	-5.103E-03	8.441E-03	5.042E-04	3.658E-01	1.030E-01
E_{rr}	2.866E-03	1.489E-01	4.699E-04	4.850E-01	6.280E-02
$E_{\theta\theta}$	5.331E-03	4.121E-01	1.372E-03	5.510E-01	4.619E-02
$E_{r\theta}$	2.211E-03	3.684E-01	3.278E-04	7.031E-01	1.914E-02
$E_{\theta z}$	3.387E-03	1.785E-03	-4.284E-05	8.751E-01	3.279E-03
E_{rz}	4.406E-04	4.075E-01	1.050E-04	5.765E-01	4.065E-02
E_{max}	6.568E-03	3.284E-02	1.146E-03	2.442E-01	1.649E-01
S_{max}	7.686E-03	4.684E-03	6.594E-04	3.795E-01	9.761E-02
ALD	1.140E+00	1.148E-01	-2.847E-02	9.047E-01	1.905E-03

Table S8: Linear regression model for the effect of TLPD (IOP-ICP) change (mmHg) on the LC strain and ALD change (μm) response ($n = 10$ eyes of 7 patients). Positive ALD change is in the posterior direction.

Row	Intercept (c_0)	p-val c_0	TLPD Change (c_1)	p-val c_1	R^2
E_{zz}	-3.774E-03	5.134E-01	-5.579E-05	8.977E-01	2.197E-03
E_{rr}	-1.116E-03	8.655E-01	3.589E-04	4.813E-01	6.381E-02
$E_{\theta\theta}$	-7.612E-03	7.360E-01	1.152E-03	5.076E-01	5.671E-02
$E_{r\theta}$	-5.217E-03	5.238E-01	6.163E-04	3.314E-01	1.179E-01
$E_{\theta z}$	4.754E-03	1.026E-01	-1.118E-04	5.850E-01	3.890E-02
E_{rz}	-9.544E-04	5.992E-01	1.200E-04	3.929E-01	9.251E-02
E_{max}	-2.293E-03	8.093E-01	8.085E-04	2.815E-01	1.429E-01
S_{max}	1.516E-04	9.827E-01	6.569E-04	2.383E-01	1.688E-01
ALD	3.183E+00	1.797E-01	-1.636E-01	3.503E-01	1.095E-01
